# Active States During the Reduction of CO_2_ by a MoS_2_ Electrocatalyst

**DOI:** 10.1021/acs.jpclett.2c03942

**Published:** 2023-03-27

**Authors:** Khagesh Kumar, Sasawat Jamnuch, Leily Majidi, Saurabh Misal, Alireza Ahmadiparidari, Michael A. Dato, George E. Sterbinsky, Tianpin Wu, Amin Salehi-Khojin, Tod A. Pascal, Jordi Cabana

**Affiliations:** † Department of Chemistry, University of Illinois at Chicago, Chicago, Illinois 60607, United States; ‡ ATLAS Materials Physics Laboratory, Department of Nano and Chemical Engineering, University of California, San Diego, La Jolla, California 92023, United States; § Department of Mechanical and Industrial Engineering, University of Illinois at Chicago, Chicago, Illinois 60607, United States; ∥ Advanced Photon Source, Argonne National Laboratory, Argonne, Illinois 60439, United States

## Abstract

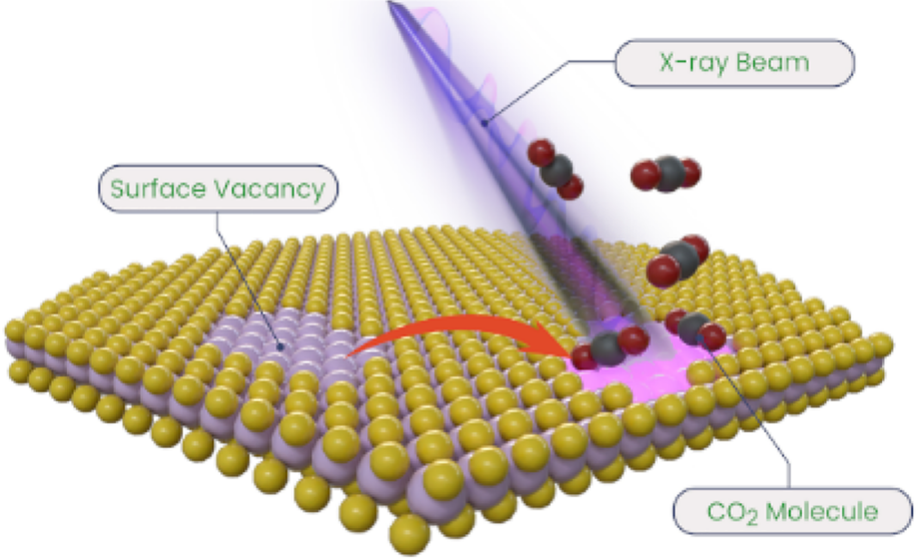

Transition-metal dichalcogenides (TMDCs) such as MoS_2_ are Earth-abundant catalysts that are attractive for many chemical processes, including the carbon dioxide reduction reaction (CO2RR). While many studies have correlated synthetic preparation and architectures with macroscopic electrocatalytic performance, not much is known about the state of MoS_2_ under functional conditions, particularly its interactions with target molecules like CO_2_. Here, we combine *operando* Mo K- and S K-edge X-ray absorption spectroscopy (XAS) with first-principles simulations to track changes in the electronic structure of MoS_2_ nanosheets during CO2RR. Comparison of the simulated and measured XAS discerned the existence of Mo-CO_2_ binding in the active state. This state perturbs hybridized Mo 4d-S 3p states and is critically mediated by sulfur vacancies induced electrochemically. The study sheds new light on the underpinnings of the excellent performance of MoS_2_ in CO2RR. The electronic signatures we reveal could be a screening criterion toward further gains in activity and selectivity of TMDCs in general.

Carbon dioxide (CO_2_) is one of the biggest drivers of climate change^1^ and, as a result, the primary target in the push to reach net-zero carbon emissions by 2050. A carbon-neutral, or even negative, cycle demands viable approaches to capture and convert CO_2_ into compounds with added value.^2,3^ Consequently, a great deal of research is ongoing toward means to promote the CO_2_ reduction reaction (CO2RR).^4,5^ Layered transition metal dichalcogenides (TMDCs) of the general formula MX_2_ (M = 4d or 5d metal, X = S–Te) constitute a class of compounds attracting interest for this purpose thanks to high activity and physicochemical tunability.^6^ Beyond CO2RR, TMDCs have shown broad catalytic activity toward other reactions, such as oxygen reduction,^7^ hydrodesulfurization,^8,9^ and hydrogen evolution (HER).^10,11^ Among TMDCs, MoS_2_ has the most established track record as a CO2RR electrocatalyst,^12,13^ thus serving as a good model system to uncover fundamental underpinnings of function that may apply to TMDCs as a class of materials.

MoS_2_ shows a high selectivity toward reduction of CO_2_ to CO at low overpotential when an ionic liquid cocatalyst is present in the aqueous electrolyte.^12^ Increases in electrocatalytic activity of MoS_2_ for CO2RR have been correlated with the existence of nanoscale defect sites, such as edges and S vacancies.^14,15^ Beyond CO2RR, the electrochemical generation of S vacancies in MoS_2_ prior to conducting HER has been proposed to enhance its electrocatalytic activity.^16−18^ Conventional correlations between synthetic control and macroscopic CO2RR performance in the literature do not directly probe mechanistic causality. Such outcome demands computational studies, usually performed using quantum mechanical electronic structure calculations, employing density functional theory (DFT).^19−21^ These studies have, in turn, revealed that sulfur vacancies are associated with electronic states within the bandgap of MoS_2_ that are centered at Mo,^16^ which could be leveraged as an active site for CO2RR.^21^ While these calculations reveal important insights into the scaling relationships of different molecular intermediates of CO2RR at defect sites,^19−21^ the electronic features that define the interaction between them and the MoS_2_ electrocatalyst in the active state remain unclear. Broadly speaking, unambiguous experimental verification of the proposed computational mechanisms of CO2RR by MoS_2_ has not been articulated, largely owing to the mismatch in length and time scale between theory and experiments.

In this contribution, we follow the *evolution* of the electronic structure in nanostructured MoS_2_ electrocatalysts during CO2RR using *operando* X-ray absorption spectroscopy (XAS). We probed both the ligand and metal centers under functional CO2RR conditions and simulated the corresponding spectroscopic signatures using first-principles calculations. Thus, we report direct spectroscopic evidence of CO_2_ binding to Mo at sulfur vacancies, which perturbs the hybridization between S 3p and Mo 4d states. The spectroscopic signatures of the active state are consistent with the reduction of Mo, revealing its role in mediating charge transfer to CO_2_. This study provides direct mechanistic evidence of the critical role of the generation and binding at sulfur vacancies for the reduction of CO_2_, pinpointing the importance of Mo–S hybridization in the design of electrocatalysts with high activity for the conversion of this greenhouse gas.

Critically, since XAS inherently averages over a large volume ensemble, to maximize the contribution from the catalytically active states to the signal we synthesized MoS_2_ nanosheets where each particle has a high surface area to volume ratio, following established synthetic protocols in the literature.^22^ Detailed synthetic methods are described in the Supporting Information.The product of the synthesis had an XRD pattern and TEM nanostructure similar to these previous results, and consistent with the formation of MoS_2_ without long-range coherence along the *c* axis beyond a few monolayers (see Figure S1).^22,23^ The activity and selectivity of gas production of the MoS_2_ electrocatalyst were analyzed in 1 M choline chloride and 1 M KOH hybrid electrolyte using linear sweep voltammetry (LSV) and differential electrochemical mass spectrometry (DEMS), respectively (see Figure S2). During DEMS, CO was shown to be the major gas product with an onset potential below −0.4 V_RHE_, while H_2_ evolution remained negligible and sharply increased at high overpotentials and below −1.2 V_RHE_. This electrocatalytic behavior toward CO2RR was consistent with previous reports in the literature.^12,24^

*Operando* XAS measurements were performed at various potentials to study the evolution of the electronic and chemical states at both Mo and S centers, using cells made in-house (Figure S3). The Mo K-edge XAS of MoS_2_ arises from a dipole-allowed transition from 1s → 5p orbitals, then further to continuum states, and is a good measure of the formal oxidation state.^25^ No pre-edge features could be resolved above the background, consistent with previous observations.^26,27^ Spectra were recorded at *E*_RHE_ = −0.58, −0.77, −1.02, and −1.24 V_RHE_ (Figure S4), as well as under open circuit voltage before (OCV) and after (post-OCV) the electrochemical process (Figure 1).

**Figure 1:**
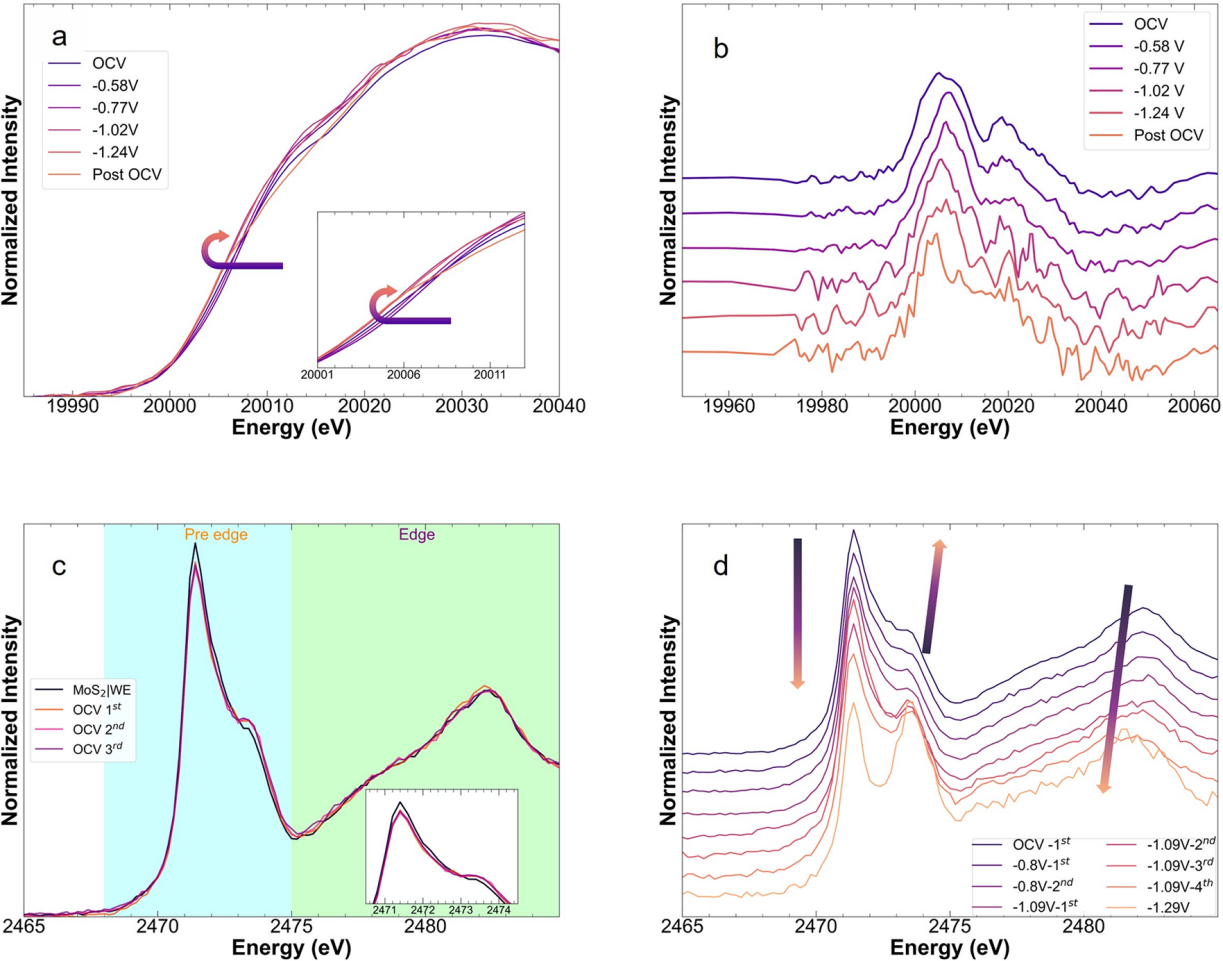
*Operando* K-edge XAS of MoS_**2**_ nanosheets. (a) Mo K-edge XAS progression following OCV → negative potentials → Post OCV. (b) First derivative of the Mo K-edge XAS under *operando* conditions in a. (c) S K-edge XAS of MoS_2_|WE and under OCV conditions, and (d) recorded under *operando* conditions. Colored arrows provide a guide to the eye for the general trends observed with decreasing potential.

During CO2RR, we found that the position of the absorption edge shifted to a slightly lower energy with electrode potential (Figure 1a), indicating that the formal state of Mo was reduced. The small shift of ∼0.9 eV was more apparent in the first derivative of the spectra (Figure 1b) and by extracting the center of gravity of the edge jump through spectral integration (Figure S5).^28^ This observation was consistent with studies of intercalation compounds of MoS_2_, where subtle shifts were found even for formal oxidation states ranging from +2.7 to 4.^29,30^ The redshift was accompanied by a change in the shape of the absorption edge, with the shoulder at 20007 eV gaining intensity as the potential decreased. Such changes in line shape can be attributed to changes in the local coordination environment of Mo.^31^ The average position of the edge was restored upon relaxation after electrocatalysis (see post-OCV in Figure 1a and Table S1). However, the shape of the white line was slightly different than the initial OCV spectrum, which points to subtle irreversible changes to the Mo coordination. We investigated the role of the local environment and morphology in modulating the Mo K-edge XAS by performing first-principles calculations, using an efficient approach that incorporates many-body excitation physics (see details in sections 12–14 of Supporting Information). These calculations confirmed that the Mo K-edge XAS was very sensitive to its local environment, evident by comparing the line-shape and peak positions of the simulated spectrum obtained from the ideal static crystal (0 K) to the disorder introduced by simply ensemble averaging from an AIMD trajectory at a finite temperature (300 K, Figure S6a).

Valence d states play a central role in transition metal catalysis.^32^ These states cannot be interrogated at the Mo K-edge due to selection rules, but they are detectable at the S K-edge via hybridization. Of note, the S K-edge XAS can be divided into two regions of interest: pre-edge and main edge, below and above 2475 eV, respectively (Figure 1c). The pre-edge results from a dipole allowed transition of an electron from 1s → S 3p–Mo 4d manifold, so its evolution is most relevant in this work. in turn, the main edge results from transition to S 3p–Mo 5s/5p hybridized orbitals, followed by higher transitions and, ultimately, photoionization.^29^ The *operando* S K-edge XAS of MoS_2_ was recorded on a MoS_2_ without electrolyte on the working electrode (referred as MoS_2_|WE), and with electrolyte at OCV and *E* = −0.80, −1.09, and −1.29 V_RHE_ (Figure 1d). The spectrum collected for MoS_2_|WE (Figure 1c) showed a sharp peak at 2471.4 eV and a shoulder at 2473.4 eV, the pre-edge peak and shoulder intensity ratio and features at the edge are consistent with previous findings in the literature.^26,27,29,33,34^ Here, and, in contrast to the Mo K-edge, our simulated XAS showed that finite temperature distortions have a muted effect on the S K-edge XAS, with both the pristine crystal and the spectra from sampling our AIMD simulation showing similar profiles (Figure S6b), which both capture well the experimental measurements.

We observed subtle changes in the peak intensity of the experimental S K-edge between the dry electrode and the first OCV scan with electrolyte saturated with CO_2_ (Figure 1c). No further changes were observed after three scans at OCV, indicating the absence of beam damage. Upon cathodic biasing, the spectra evolved even during some potentiostatic holds. To capture these changes, the scans at a fixed potential are shown as they were collected (Figure 1d). In general, with increasing cathodic bias, the intensity of the pre-edge peak at 2471.4 eV decreased, and the intensity of the shoulder at 2473.4 eV increased. These changes are a clear indication of the perturbation of the S 3p–Mo 4d manifold during CO2RR. Moreover, the spectra showed a shift in the main edge to a lower energy, manifested in an increase in the intensity of a shoulder at 2478 eV, and a change in energy from 2482.5 to 2481.5 eV in the position of the apex (see arrows in Figure 1d). The changes at the main edge are indicative of a decrease in the effective nuclear charge of S (*Z*_eff, S_), reflecting a gain in electron density around S.^27,29^ The S K-edge redshifts followed a similar trend to the Mo K-edge, both being most pronounced around ∼ −1 V_RHE_ (Table S1) and suggesting that electronic changes correlate with increased benchmarked activity (Figure S2). This observation indicates that S participates in the charge compensation mechanism induced during electrocatalysis.

To further elucidate the physics behind the observed spectral changes, we simulated the *operando* XAS using snapshots obtained from AIMD simulations at 300 K. Here, we employed DFT to simulate the electronic structure of different MoS2 intermediates, coupled with the effective screening media method (ESM) to simulate the electrochemical effects. The XAS simulation were performed within a multideterminant, delta self-consistent field approach, Further simulation details can be found in the Supporting Information (12–22). In both Mo and S K-edge, we found that the simulations of the pristine structures under negative bias were similar to the OCV spectrum (Figure S7), suggesting that charging alone does not significantly alter the unoccupied states of MoS_2_. We note that any discrepancies between experimental and simulated spectra intensities can result from several factors, including the limited ability to normalize the experiments, due to the requirement for background subtraction, whereas the calculation does not have any background signal. Nevertheless, in our computational approach, these differences are self-contained, which allows us to quantitatively compare spectra changes across different structures, producing relative intensities that compare well with experiments. We then explored the effect of S vacancies on the S K-edge XAS (Figure 2a), going from isolated single sites to increasing concentrations of S vacancies. To gain insights into the nature of the corresponding excited state, we considered the electron density around the excited atom with and without the excited electron present. We refer to this as the “differential excited state charge density”, which can be interpreted as representative of the orbital or state that the excited electron occupies in the lowest energy transition.^35^ Our calculations indicate that for the pre-edge peak, the excited electrons have S p and Mo d hybrid symmetry (Figure 2b), as expected. The excited electron was also fairly delocalized, with significant intensity on neighboring Mo atoms and spanning neighboring MoS_2_ layers. The consequence to the spectra of this delocalized excited electron is a broad (reduced intensity) pre-edge at 2471 eV, and a relative increase in the intensity of the shoulder at ∼2473 eV, with increasing vacancy concentration (Figure 2c). This result is consistent with our experiments, so we conclude that the observed increase in the experimental peak intensity at 2473.4 eV vs 2471.4 eV is a signature of significant S defect formation. The observations match with the reported lowering of S K pre-edge intensity with no change in the position upon *in situ* S vacancy generation in monolayer MoS_2_.^36^

**Figure 2:**
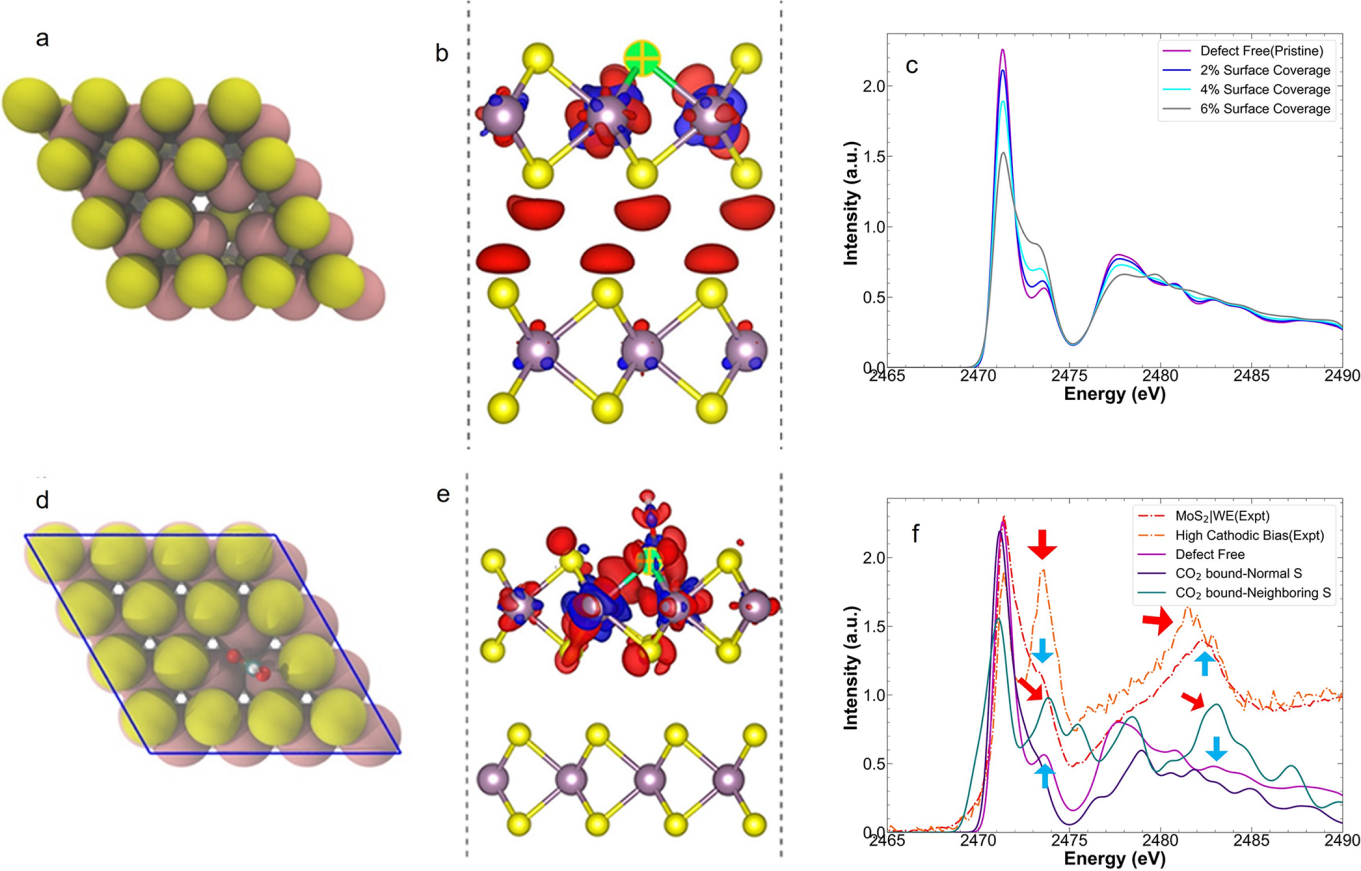
Simulated spectroscopic signature of the binding of CO_2_ to MoS_2_. (a) Atomic representation of a single anionic vacancy (blank site) in MoS_2_. The Mo and S atoms are shown as pink and yellow spheres, respectively. (b) Representative differential excited state charge density of the S 1s → first conduction band excited electron. The dashed vertical lines indicate the unit cell boundaries. We adopt the convention that the increase in the density is colored blue, while reduction is colored red. The excited S atom is indicated by the green crossed symbol. (c) Simulated S K-edge XAS as a function of concentration of S vacancies. (d) Atomic structural representation of CO_2_ bound to Mo using a S-vacancy site in MoS_2_. The O and bound C atoms are shown as red and brown spheres, respectively. (e) Representative excited electron charge density. (f) Comparison of the S K-edge XAS, calculated using the defect-free MoS_2_ structure (purple line) and the structure with a CO_2_ bound to a S vacancy site (blue and teal). The experimental XAS of the MoS_2_ measured in the MoS_2_|WE and at high cathodic bias (−1.29 V_RHE_) are shown as a reference (red and orange dotted lines, respectively). The red arrows indicate the correspondence between the simulated XAS of the S atoms neighboring a CO_2_-bound vacancy and the high cathodic experiments, while the blue arrows indicate the correspondence between the simulated pristine MoS_2_ structure and the initial experimental measurement in the MoS_2_|WE.

Our simulated spectra also reveal significant modulation in the S K-edge XAS after CO_2_ binds to Mo atoms near the S vacancy (Figure 2d).^19−21^ This effect is especially noticeable when considering the spectrum of the S ions adjacent to the vacancy occupied by CO_2_, since the associated excited electron state is hybridized and spatially delocalizes over several neighboring atoms, including the CO_2_ molecule that binds to the Mo at the S vacancy (Figure 2e). Here, the excited electron state is more localized within the MoS_2_ layer. It leads to an increased intensity in both the shoulder of the prepeak and the main edge peak. We obtained further insights by projecting the ground state electronic density of states of the S atoms, which showed that the conduction band of sulfur atoms neighboring the reaction site is red-shifted to lower energy (Figure S8) and has a dominant p-orbital character. Furthermore, the hybridization between neighboring CO_2_ lowers the energy of the states in the conduction band and manifested as a slight redshift and broadening in the first pre-edge (Figure 2f). Overall, we summarize that when CO_2_ binds to the S vacancy, this results in (i) suppression of the pre-edge peak near 2471.4 eV, (ii) an increase in the pre-edge shoulder near 2473.4 eV, due to contributions from sulfur atoms neighboring the bound CO_2_, and (iii) an increase in the main-edge peak near 2478 eV (Figure 3 and Figure S9). All these trends are in very good agreement with our experimental measurements at high cathodic bias.

**Figure 3:**
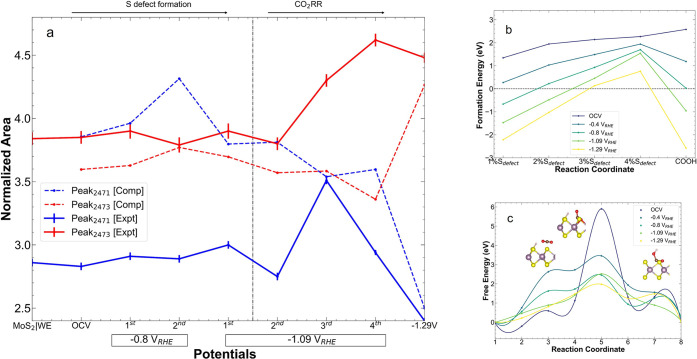
Mechanism of CO_2_ binding to MoS_2_. (a) Calculated area of the two pre-edge peaks, corresponding to 2471.4 and 2473.4 eV in the pristine spectrum from a MoS_2_|WE, extracted from fits of the S K-edge shown in Figure S10. (b) Calculated defect formation energy of vacancy (with S1 ≥ 1% to S4 ≥ 4% vacancy) and CO_2_ bound MoS_2_ as a function of bias voltage (0, −0.40, −0.80, −1.09, and −1.29 V_RHE_). (c) Reaction free energy of CO_2_ binding to a structure with 3% S vacancies, for the neutral and charged (−0.4, −0.8, −1.09, and −1.29 V_RHE_) cases, from nudged elastic band calculation. Three representative molecular structures along the reaction coordinate are shown in the inset.

Our XAS results so far now position us to propose that MoS_2_ undergoes a process of reduction in the active state during CO2RR, which shifts both Mo and S K-edges to lower energy. However, we hypothesize that such a reduction is part of a more complex process that requires significant bias as a driving force, initially to create S defects as binding sites, and then to overcome the CO2RR barrier to react at them. We first provide evidence for this hypothesis directly from our experimental S K-edge XAS measurements and then show how this is consistent with our *operando* XAS simulations.

Considering first the experimental data, we fitted the spectra with pseudo-Voigt and Gaussian functions in the pre-edge and main-edge regions, respectively^37,38^ (Table 1 and Figure S10). At lower potentials, the spectral area of the pre-edge peak at 2471.4 eV was initially constant, with a subtle increase in width. However, we found a marked increase in the peak area at −1.09 V_RHE_, followed by a sharp decrease at even more reducing conditions (Figure 3a). In contrast, the shoulder feature at 2473.4 eV gained intensity and shifted to a higher energy at the same negative potentials. At low cathodic potentials and with time (2nd scan at *E*_RHE_ = −1.09 V_RHE_ onward), this evolution in fact led to two well-separated peaks. The most pronounced shift of the main edge toward lower energies was also observed at these most reducing potentials (Figure 1d). Overall, these effects further point at S playing an active role in CO2RR, by taking electron density and via the reorganization of the S 3p-Mo 4d manifold that act as active states.^32^

**Table 1:** Results of the Fits of Operando S K-Edge XAS^a^

sample	Position_2471_	Position_2473_	Position_Edge_	δ_2471_ (eV)	δ_2473_ (eV)	Area_2471_	Area_2473_
MoS_2_|WE	2471.4	2473.1	2482.3	10.9	9.2	2.86 ± 0.03	3.84 ± 0.05
OCV	2471.4	2473.25	2482.2	10.8	9.0	2.83 ± 0.03	3.85 ± 0.05
–0.80 V_RHE_ 1st	2471.4	2473.32	2482.2	10.8	8.9	2.91 ± 0.03	3.90 ± 0.06
–0.80 V_RHE_ 2nd	2471.4	2473.32	2482.2	10.8	8.9	2.89 ± 0.03	3.79 ± 0.06
–1.09 V_RHE_ 1st	2471.4	2473.33	2482.1	10.8	8.8	3.00 ± 0.03	3.90 ± 0.06
–1.09 V_RHE_ 2nd	2471.4	2473.41	2482.1	10.8	8.7	2.75 ± 0.03	3.80 ± 0.05
–1.09 V_RHE_ 3rd	2471.4	2473.45	2481.9	10.8	8.5	3.51 ± 0.03	4.30 ± 0.05
–1.09 V_RHE_ 4th	2471.4	2473.41	2481.7	10.8	8.3	2.94 ± 0.02	4.62 ± 0.05
–1.29 V_RHE_	2471.4	2473.6	2481.5	10.1	7.9	2.40 ± 0.02	4.48 ± 0.03

a The fits can be found in Figure S10. δ_2471_ and δ_2473_ are the energy differences between pre-edge fit peak positions and the edge; Area_2471_ and Area_2473_ refer to area under the corresponding pre-edge fits (see Figure S10).

The spectral reorganization observed at the pre-edge of S implies changes in the energy of the S 3p-Mo 4d unoccupied states. Using the main S K-edge as an estimate of the photoionization threshold, its separation relative to the pre-edge has been proposed as an estimate of the ligand field destabilization of the S 3p-Mo 4d antibonding states.^39,40^ Borrowing conventions from the literature,^39^ we defined δ = Energy_main-edge_ – Energy_pre–edge_,^39^ with δ_2471_ and δ_2473_ referring, respectively, to the sharp feature at 2471.4 eV and its shoulder at 2473.4 eV in the OCV spectrum (see Table 1). Both δ_2471_ and δ_2473_ decreased with potential, suggesting that the antibonding orbitals get progressively destabilized, shifting closer to the photoionization threshold. However, the change was most pronounced for δ_2473_, inducing an increased splitting of the two pre-edge peaks, from 1.8 eV at OCV to 2.2 eV at −1.29 V_RHE_. Close observation of the spectra reveals that the evolution of δ_2471_ is driven only by the redshift of the main-edge, in contrast to δ_2473_, where both the main-edge and the pre-edge peak approach each other (Figures 1d, S10).^27,29^

It is instructive to compare the behavior of the XAS of MoS_2_ under cathodic CO2RR with the charge compensation when it undergoes reduction via electrochemical Li intercalation.^27,29^ The evolution of the main edge in the S K-edge XAS is similar in both reactions.^27,29^ However, while the formation of Li_*x*_MoS_2_ induces a *decrease* in intensity of *both* pre-edge peaks, without any obvious changes in position,^27,29^ during CO2RR, and despite other notable changes, the total intensity of the pre-edge did not change significantly (Table 1). This behavior further supports that the hybridization between S 3p and Mo 4d states change concurrent to reduction during electrocatalysis, as seen in molecular systems.^37,38^ The comparison thus suggests a different mechanism and possibly changes in the electronic states during CO2RR electrocatalysis than present in lithiation, where a classical process of reduction operates.

To validate our electrochemically driven vacancy formation and subsequent CO_2_ binding hypothesis, we utilized additional first-principles simulations. First, we considered the role of electrochemistry in stabilizing S vacancies and found that the formation energies of S vacancies and CO_2_-vacancy complexes are all more favorable at negative potentials. For instance, the formation energy for the structure with 4% defects lowered from 2 eV at OCV to ∼1 eV at −1.09 eV, to ∼0.5 eV at −1.29 eV (Figure 3b). This is due to the presence of midgap states arising from the S vacancies; these states act as a new LUMO situated at lower energy and are filled during the electrochemical reaction leading to a relatively more stable system. In fact, our calculations indicate a thermodynamically accessible formation energy for a 3% vacancy structures at −1.09 eV. Moreover, assuming a 3% vacancy structure, we found that the barrier for CO_2_ insertion into a MoS_2_ vacancy dramatically reduced with increased bias voltage, from >6 eV at OCV to <2 eV at −1.29 eV (Figure 3c). The increase in stability of these vacancy sites as the active sites for CO_2_ binding and a favorable pathway due to negative potential together promote greater CO2RR. Our calculation shows good agreement with recent work where basal plane MoS_2_ S vacancies are favorable to CO2RR.^41^

We then considered the effect of CO_2_-bound vacancies on the simulated XAS. As noted previously, the simulated S K-edge XAS of the pristine system is relatively insensitive to applied bias and the XAS of S atoms that are second nearest neighbor to the vacancy or binding site showed similar spectral features with pristine MoS_2_. Therefore, we considered the simulated S K-edge XAS of a series of optimized structures, following our proposed mechanism, that starts from the pristine MoS_2_ surface (from 0 to −0.8 V), followed by nearest neighbor S vacancy structures of increasing size (from −0.8 to −1.0 V) and, last, CO_2_-vacancy structures (from −1.0 to −1.3 V). To further verify that the experimental XAS spectrum corresponds to CO_2_ bound to S vacancies (and not any other bound CO2RR intermediates) during the electrocatalysis, we simulated the XAS response of other possible bound intermediates along the CO2RR path (Figure S11). The energy axis of each different compound is calibrated according to section 16 of the Supporting Information. Notable, during electrocatalysis, we observed experimentally a significantly suppressed pre-edge peak at ∼2471 eV, and growth of a second feature at higher energy. These features were not present in any of the other intermediates studied: *COO, *CO, and *CH_2_ (Figure S11), which all present unambiguous peaks below 2469 which would be clearly visible, if present. However, no signals at such low energy were observed experimentally. The simulated spectral signature only matches well with the presence of *COOH, which is the first intermediate along the reaction path after CO_2_ binding. The spectra starts to differ greatly along the reaction path once the local environment around S changes due to the ongoing reaction, as H slowly migrates and replace O to form CH_4_. This observation also suggests that the reaction pathway involves C and O interaction with neighboring Mo centers as proposed in the literature.^42^ Similar to our experiments, we fitted the corresponding simulated pre- and main-edge features to pseudo-Voigt and Gaussian functions respectively. As shown in Figure 3a, the trends in simulated XAS peak area were in remarkable agreement with our measurements. This was achieved by direct comparison between theory and experiments, with no empirical adjustment to the simulated spectra. Thus, we conclude that the *operando* experimental XAS spectrum encodes the electrochemically driven generation and reaction of CO_2_ on MoS_2_ vacancies, which can be elucidated by first-principles calculations and spectral simulations.

In conclusion, in this work we move beyond conventional pictures of the active states during electrocatalysis where only the changes in formal oxidation state of the transition metal are considered. Indeed, our Mo and S K-edge XAS results point to the intricate role of both the metal cations and chalcogenide anions when MoS_2_ conducts CO2RR. In turn, coupling with complementary first-principles electronic structure simulations revealed that catalyst-molecule interactions modulate the electronic states of the electrocatalyst, incorporating electron injection and altered S 3p-Mo 4d hybridization physics. Comparison with simulated XAS under different scenarios reveals a detailed mechanism, first involving the formation of S vacancies electrochemically at low negative bias, followed by CO_2_ binding to the active state and electrochemical conversion at the most negative potentials. Our work reveals that the concentration of S vacancies in MoS_2_ presents unique signatures in the S K-edge XAS, providing a means to guide the design of materials where such vacancies are produced during synthesis to bypass an electrochemical step and further lower the overpotentials of CO2RR. This insight, and the use of simulated XAS to fingerprint active states generated electrochemically, refines existing models of electrocatalytic function of this class of materials. Moreover, such combined experiment/theory studies at the anion K-edge open up the possibility of providing detailed insights into a vast range of related chemical reactions.

## Supporting Information Available

Additional experimental details, materials, and methods, gas product analysis, S K pre-edge fitting, including photographs of *operando* setup. Computational methods from DFT ground state, vacancies formation energy, and nudged elastic band calculations, AIMD and XAS simulations (PDF)
VASP poscars for the optimized structures (ZIP)
Transparent Peer Review report available (PDF)
