# Supplementary material for: Active States During the Reduction of CO2 by a MoS2 Electrocatalyst
Source: J Phys Chem Lett. 2023 Mar 27;14(13):3222–9. doi: 10.1021/acs.jpclett.2c03942 (PMC10084464; doi:10.1021/acs.jpclett.2c03942)
Supplement: Supplementary file 1 — jz2c03942_si_001.pdf [file jz2c03942_si_001.pdf]

Active States During the Reduction of CO_2 by a MoS_2 Electrocatalyst

Khagesh Kumar,[1] Sasawat Jamnuch,[2] Leily Majidi,[3] Saurabh Misal,[3] Alireza Ahmadiparidari,[3] Michael A. Dato,[1] George E. Sterbinsky,[4] Tianpin Wu,[4] Amin Salehi-Khojin,[3] Tod A. Pascal*,[2] and Jordi Cabana*,[1]

[1]Department of Chemistry, University of Illinois at Chicago, Chicago, Illinois, 60607, United States

[2]ATLAS Materials Physics Laboratory, Department of Nano and Chemical Engineering, University of California, San Diego, La Jolla, CA 92023, United States

[3]Department of Mechanical and Industrial Engineering, University of Illinois at Chicago, Chicago, Illinois, 60607, United States

[4]Advanced Photon Source, Argonne National Laboratory, Argonne, Illinois, 60439, United States

Corresponding authors: Tod Pascal (tpascal@ucsd.edu) and Jordi Cabana(jcabana@uic.edu)

Table of Contents

Supplementary Experimental Methods

1. Materials
2. Synthesis and Characterization of MoS_2 nanosheets
3. *Operando* X-ray Absorption Spectroscopy
4. Structure of the synthesized MoS_2 Nanosheets
5. Removal of Organic Residue on MoS_2 Nanosheets
6. Calculation of the Position of the Mo K-edge using Integrated Area
7. Activity and selectivity of MoS_2 catalyst
8. Calculating Applied Potential vs RHE
9. Electrochemical performance during the *Operando* experiments
10. *Operando* Cell for Mo and S K-edge XAS
11. Fitting of the S K-edge XAS

Supplementary Computational and Theoretical Methods

12. Ground-state electronic structure calculations.
13. Ab initio molecular dynamics (AIMD) simulations.
14. Core-level X-ray response calculations.
15. Core-level XAS under applied bias and defects
16. Calibration of XAS spectra.
17. Effect of charging on MoS_2 Mo K-edge and S K-edge
18. Effect of S-vacancy and bound CO_2 on second nearest neighboring atom XAS response
19. Defect (Vacancy) energy calculations
20. Reaction mechanism by nudged elastic band calculations
21. XAS response of intermediates along reaction pathway

References

Supplementary Materials

Supplementary Experimental Methods

1. Materials.

$(NH_4)_2MoS_4$ (99.97%), Oleylamine (70%), choline chloride (> 99%), KOH (99.95%) were purchased from Sigma-Aldrich. The gas diffusion electrode was purchased from FuelCellsEtc and silicon nitride windows were purchased from Norcada.

2. Synthesis and characterization of MoS_2 nanosheets.

MoS_2 nanosheets were synthesized using a colloidal method described in the work of Altavilla *et. al.*[1] $(NH_4)_2MoS_4$ was used as a source of both Mo and S, and oleylamine was used as both solvent and capping agent. During the synthesis, the precursor was decomposed in oleylamine at 350°C to form MoS_2. After the synthesis, the product was washed with mixtures of isopropyl alcohol and hexane, followed by drying and annealing in Ar at 300°C for 8 h to remove any organic residue and clean the solid surfaces for catalysis.

Powder X-ray diffraction was performed on a Bruker D8 Advance using Cu Kα (λ_{avg} =1.54 Å) radiation. Scans were recorded from 10° to 80° (2θ). Transmission electron microscopy (TEM) was performed using a JEOL-3010 microscope operated at 300 kV.

3. *Operando* X-ray Absorption Spectroscopy.

X-ray absorption spectroscopy (XAS) was carried out at beamlines 9-BM (S K-edge) and 10-ID (Mo K-edge) of the Advanced Photon Source at Argonne National Laboratory. The Mo K-edge was collected in fluorescence mode with an ion-chamber detector in Quick-X-ray Absorption Fine-Structure (quick-XAFS) mode, and Mo foil was used for energy calibration. The S K-edge data was recorded in fluorescence mode with a 4-element silicon drift detector, in a He chamber at atmospheric pressure to reduce absorption. The energy scale of the spectra was calibrated with independent measurements of a $Na_2S_2O_3$ standard. Data reduction of the XAS spectra was performed using Athena from the Demeter package.[2] Pre-edge and post-edge backgrounds were subtracted, and the spectra were normalized with respect to edge height. The edge jump was set to 1. The S K -edge was fit with pseudo-Voigt and Gaussian functions in the pre-edge and edge features respectively, using the LMFIT package.[3]

Operando cells were made in house as shown in Figure S3, with different designs depending on the edge of interest, due to the different constraints imposed by the absorption energy. For the Mo K-edge measurements (Figure S3a), a gas diffusion electrode was drop-casted with MoS_2 nanosheets and was used as the working electrode (WE). The electrolyte was purged with CO_2 for saturation, the flow was then stopped, a potential applied under chronoamperometry and the

XAS spectra was collected. For S K-edge measurements (Figure S3b), the low energy (2500 eV) imposed the need to drop-casted with MoS_2 directly on a Si_3N_4 window (500nm thick) coated with 200 nm titanium to function as the WE. The whole electrochemical cell was placed in the He chamber to further reduce absorption by air. The electrolyte was again saturated before placing it in the cell. For all *operando* measurements, a Pt wire was used as counter electrode (CE), Ag^+/Ag was used as a reference electrode (RE) and 1 M choline chloride + 1 M KOH was used as electrolyte. A Biologic SP-50 potentiostat was used to perform chronoamperometry during the *operando* experiments. All the measurements were performed at room temperature

4. Structure of the synthesized MoS_2 Nanosheets

To understand the structure and stacking of as-prepared nanosheets, X-ray diffraction (XRD) and transmission electron microscopy (TEM) was performed (Figure S2). The XRD looked very similar to the reported pattern of mono/few layers of MoS_2,[1,4] and with an absence of $(00l)$ reflections that suggests no or weak stacking along the c axis and no match with the reported sulfur(S_8).[5] The TEM image of the as-prepared material showed crumbled sheets and no signs of stacking of the NS and the rough lateral dimension to be around 20 nm. These observations are fully consistent with previous observations in the reference studies,[1,4] indicating that the nanosheets consisted of very few layers. The XRD of as-prepared material showed a diffraction peak around 19° 2θ, it corresponds to reported diffraction peak of organic residues.[6] The annealed sample shows no peak at 19° 2θ suggesting removal of the organic residues. It is also noted that there is no occurrence of peaks around 14° 2θ for the (002) reflection, further indicating that the annealing did not lead to stacking of the NS.

5. Removal of Organic Residue on MoS_2 Nanosheets

The IR spectra was recorded for the as-prepared sample and annealed. The as-prepared sample shows presence of organic moiety and most of the peak matching with reported values for oleylamine,[7] upon annealing in Argon for 8 hrs the signal from organic moiety reduced substantially (~ 20 times) (Figure S12) suggesting the process was successful in cleaning the material from organic residue.

6. Calculation of the Position of the Mo K-edge using Integrated Area

A method of integration to produce a weighted area reported by Dau et al. was used to determine the position of the main edge of Mo.[8] In this case, $\mu_1 = 0.1$ was used to minimize contributions from the pre-edge features and $\mu_2 = 1.00$ was used to reduce effect from the strong whiteline.[8]

Table S1: Edge position of Mo K edge calculated using weighted area method[8]

Sample	Edge position (eV)
OCV	20009.5
-0.58 V_{RHE}	20009.3
-0.77 V_{RHE}	20009.1
-1.02 V_{RHE}	20008.6
-1.24 V_{RHE}	20008.8
Post OCV	20009.5

7. Activity and selectivity of MoS_2 catalyst

Linear sweep voltammetry (LSV) was performed for the MoS_2 catalyst in the *operando* cell. A Pt wire was used as counter electrode (CE), Ag^+/Ag was used as a reference electrode (RE) and 1 M choline chloride + 1 M KOH was used as the electrolyte. CO_2 was bubbled for 30 minutes into the electrolyte before the experiment. The MoS_2 catalyst was coated on a gas diffusion electrode and used as the working electrode. A current density of ~26.8 mA/cm^2 was obtained at the potential of -1.25 V_{RHE} (Figure S3a).

In separate experiments, differential electrochemical mass spectrometry (DEMS) was used to perform an online analysis of the gas product from the MoS_2 catalyst during electrochemical reduction of CO_2, using a Hiden Analytical HPR-40 system. The electrolyte was purged with CO_2 prior to attachment to DEMS. Next, the sealed system was allowed to rest for ~3 hours to stabilize before running the experiments The real-time DEMS results shown in Figure S3b were recorded during an LSV experiment (up to the potential of ~-1.35 V_{RHE}) performed on MoS_2 catalyst in a two-compartment cell with a scan rate of 1 mV/s. CO was observed to be the major gas product in this reaction, while H_2 evolution started at higher overpotentials.

8. Calculating Applied Potential vs RHE

The potential was converted to V_{RHE} using the following equation, here $V_{Ag/Ag+}$ was applied potential, pH was the pH was of the electrolyte, i is the measured current and R is electrical resistance.

$$V_{RHE} = V_{Ag/Ag+} + 0.155V + (0.0592 * pH) - i * R$$

9. Electrochemical performance during the *Operando* experiments

The electrochemical performance in the *operando* cell under chronoamperometric conditions at Mo K and Sulfur K edge (Figure S4) were recorded during the *operando* experiments. The current density was calculated using geometric area of the working electrode.

10. *Operando* Cell for Mo and S K-edge XAS

Figure S1 shows the *operando* cells used to conduct XAS at the Mo K- and S K-edge respectively. The cells were 3D printed using polylactic acid and machined from an acrylic tube, respectively, for the purpose of the experiments.

11. Fitting of the S K-edge XAS

The fits of the S K-edge are shown in Figure S10. The experimental data is shown in black. Individual pseudo-Voigt functions were used for the pre-edge, and they are shown in dashed blue and red colors. Gaussian functions were used for the main edge, which are shown in green. The overall sum of features is displayed in violet. The shaded blue and red peak areas corresponds to sharp feature at 2471.eV and the shoulder at 2473.4 eV respectively. $Area_{2471}$ and $Area_{2473}$ denotes area under the corresponding pre-edge fits and $\delta 2471$ and $\delta 2473$ (Figure S10a), shows the difference in energy of the respective pre-edge fits from the edge.

Supplementary Computational and Theoretical Methods

12. Ground-state electronic structure calculations.

First-principles DFT within the Quantum-ESPRESSO package[9] was used to simulate the electronic states of MoS_2.[10] We first optimized a hexagonal unit-cell of MoS_2 with lattice constants a = b = 3.161 Å, c = 12.295 Å, α = β = 90° and γ = 120°, which was used as a model system. Ultra-soft pseudopotentials[11] were employed under periodic boundary conditions and a plane-wave basis set with a kinetic energy cut of 30 Ry. The exchange-correlation by Perdew, Burke and Ernzerhof was used.[12] Grimme's DFT-D3 corrections was included to account for the missing van de Waals interaction in PBE. We employed the effective screening medium method (ESM) to accurately simulate the electronic states of MoS_2 electrode under bias voltage.[13] The effective screening media method is a framework developed for first-principles simulating of electrified interfaces, where the system Hamiltonian includes an additional term representing the interaction between electronic charge and an imaginary dielectric screening medium. Thus, a specific bias potential can be simulated by a Green's function of the charge (the charge – number of electrons – and the potential are conjugate pairs). The applied voltage employed in the simulation is the result of electrostatic potential difference between MoS_2 and the ESM.

13. Ab initio molecular dynamics (AIMD) simulations.

Molecular dynamics simulation of MoS_2 was performed within Vienna Ab initio Simulation Package (VASP).[14] A 3x3x1 MoS_2 supercell (using the optimized unit cell in 2.2.1) was propagated for at least 5ps at 300K, with the temperature controlled by a Nose-Hoover thermostat.[15] All simulations used a Monkhrost 2x2x2 k-point grid.[16] Snapshots of the system, representing the instantaneous atomic position were extracted every 100 fs during the last 2 ps of AIMD, and used as input to simulate the XAS.

14. Core-level X-ray response calculations.

We employed the delta-self-consistent-field (ΔSCF) method[17] to simulate the XAS response of the system. We first generated the Kohm-Sham orbitals from two self-consistent fields, representing ground and the core-excited states. In particular, the core-excited electronic states were modeled using a constrained electronic configuration, realized by generating a new pseudopotential with a core-hole, i.e. with a $1s^1$ configuration, representing K-edge excitation.[18] The resulting electronic states was then used as input for constructing a Slater determinant of the overlap matrix between the ground and core-excited states, according to the MBXAS formalism.[19] This approach has been shown to be efficient (so that we can use multiple snapshots from our MD trajectory), with an accuracy comparable to more elaborate levels of theory. We converged each spectrum by sampling a 5x5x5 k-point grid within the Brillouin zone.

15. Core-level XAS under applied bias and defects.

The energy levels and character of both the occupied and unoccupied states, especially those near the Fermi level, will change due to the injection of electrons. As a practical scheme for realizing the effect of this physics on the XAS, we devised an approach to self-consistently generate the ground state and core-excited state in the presence of a perturbative external field

meant to mimic an applied bias. Specifically, we employed the electronic screening method,[13] a Green's function approach where the applied bias is introduced as an additional term in the electronic Hamiltonian. We also explored the XAS response from MoS_2 with S-vacancies, both at single vacancy sites (representing a ~3% defect level) and at agglomerated vacancy sites (a ~12% defect level). Finally, we investigated the MoS_2 with a bound CO_2 molecule at one of these sites, according to the reaction pathway proposed by Kang and co-workers.[20]

16. Calibration of XAS spectra.

The use of pseudopotentials in our calculations means that we lose the absolute reference state from which we can base our excitation energies, as would be present in an all-electron calculation. Thus, each spectra needed to be properly calibrated for unambiguous comparisons. In this work we apply two independent calibration schemes. First, we obtained an absolute energy shift that was applied to all spectra by matching the main edge of our simulated spectra for pristine MoS_2 (2467.2 eV for sulfur and 20000.1 eV for molybdenum) to the experimental measurement of the dry sample. Next, we apply to the formation energy formulism of Prendergast and coworkers[21] to compare simulated spectra of system comprising different unit cells, number of atoms and applied bias. Previous work has shown that this approach is accurate in predicting the main peak position of various lithium compounds to within 0.1 eV of the experiments.[22]

17. Effect of finite temperature on XAS response

Intrinsic vibration within material can lead to differences between observed experimental result and theoretical calculation, usually due to finite temperature broadening. Thus, the simulated results can be improved by performing additional sampling, beyond the pristine crystal structure, realized here by extracting snapshots from AIMD simulations.

We performed AIMD simulation on 3x3x1 supercell of 2H MoS_2 where the electrons are described with DFT at the PBE level of theory using the Vienna Ab initio Simulation Package (VASP).[14] We evolved the system forward in time for at least 100ps, in the constant particles (N), constant volume (V) and constant temperature (T) ensemble (i.e., the canonical or NVT ensemble). We constrained the temperature of the system to be 300K by means of a Nose-Hoover thermostat with a time relaxation constant of 20fs, and included empirical dispersion corrections to account for the missing van der Waals interactions in PBE by means of the Grimme DFT-D3 approach.[23] The resulting final 50 ps equilibrated trajectories of were then used for analysis.

During our AIMD simulations, we observed that the Mo atoms displacement was 0.1161 ± 0.0461 Å while the S atoms displacement on average was higher at 0.1442± 0.0616 Å. We also quantified any asymmetry in the calculated displacement and found that the in-plane motions between the two species were identical, however the out of plane motions are asymmetric. Thus, in order to properly sample this asymmetry, we took snapshots (atomic positions) of the system 5 ps apart, which we verified lead to spectral convergence.

18. Effect of charging on MoS_2 Mo K-edge and S K-edge

We employed the effective screening media method[13] to simulate an MoS_2 electrode under applied bias. We found that the *operando* bias voltage did not significantly change the spectral line shapes of the pristine sample at reasonable bias (i.e., <5V). We then looked into an extreme, where significant amount of charge (corresponding to ~ 7.5 V, far beyond what is practical in

experiments) was added into the system. The resulting high electron injection led only to a slightly suppressed first peak in the sulfur K-edge and Mo K-edge, and a redshift in the Mo K-edge peak. Thus, we conclude that charging of the system would not provide drastic change to the spectral lineshape, which suggests that they instead arises from other (possibly chemical) factors.

19. Effect of S-vacancy and bound CO_2 on second nearest neighboring atom XAS response

We verified that spatial extent of the XAS response contained contribution from the immediate local environment of the active site by calculating the XAS of the second nearest neighbor atoms. The results show that the spectral lineshape and feature of these atoms greatly resembles that of the pristine structure.

20. Defect (Vacancy) energy calculations

We used the energy and potential from our ground state electronic structure calculations to determine the defect formation energy E_f of defect X in charge state q, according to the approach of Komsa, which was later adopted by Huber and coworkers:[24,25]

$$\boldsymbol{E_f[X^q] = E[X^q] - E_{host} + E_{corr} - \sum n_i\,\mu_i + q(\varepsilon_F + \varepsilon_v + \Delta v)}$$

where $E[X^q]$ and E_{host} are the total energies of defective and pristine structure respectively, E_{corr} is the image-charge correction energy to account for long-range coulombic interaction, n_i is the number of species i (it is positive when added and negative otherwise) and μ_i is the chemical potential of the species i. The charge q term is the cost of adding or removing an electron with respect to the potential set at the Fermi energy ε_f and the energy of valence band maximum ε_v. The Δv term was used to align the potential between the bulk and defect states.

21. Reaction mechanism by nudged elastic band calculations

We performed nudged elastic band calculation (NEB)[26,27] of the MoS_2 reaction pathway to verify the transition state stability with and without bias voltage. The result shows that the reaction is possible after bias voltage is applied to the system with prohibitively large energy barrier for absence of driving voltage.

22. XAS response of intermediates along reaction pathway

We simulated S K-XAS spectra corresponding to the CO_2 binding to S vacancies during the electrocatalysis and the spectral response to possible bound intermediates along the reaction path.

Supplementary Figures

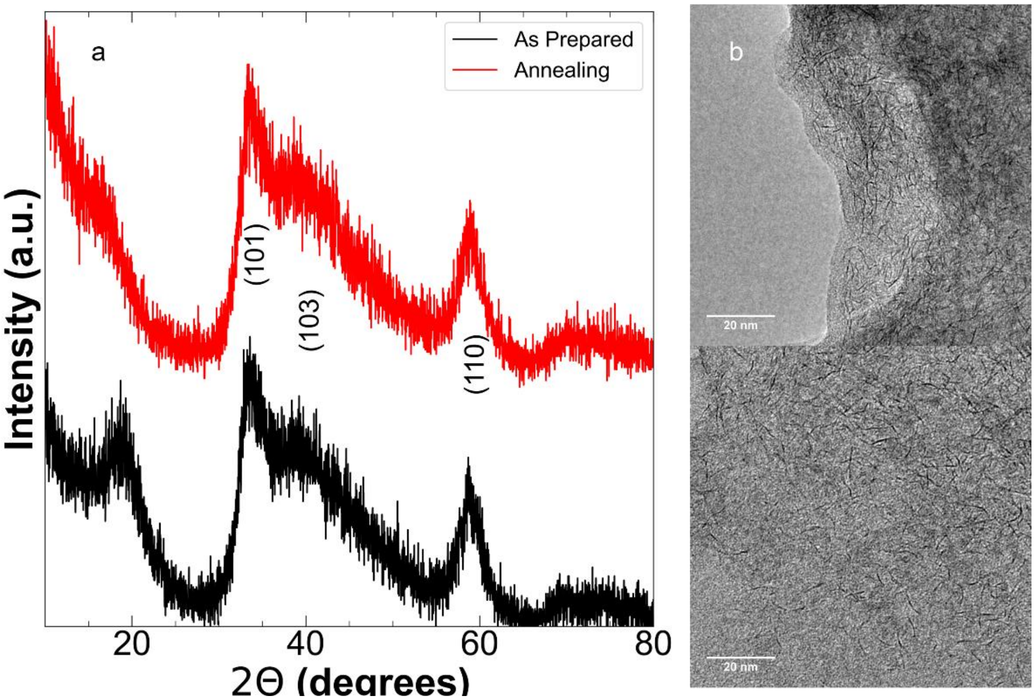

Figure S1: (a) XRD of as-prepared and annealed MoS_2. The removal of the peak at 19°, 2θ, suggests the removal of organic residues by annealing. The absence of $(00l)$ peaks suggest that there is no long-range coherent stacking of sheets. (b) TEM of as-prepared MoS_2.

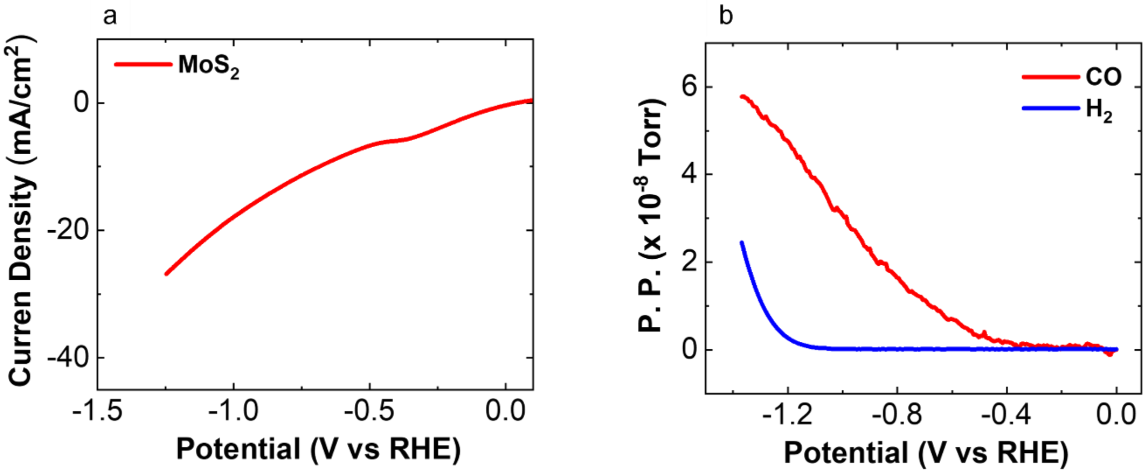

Figure S2: (a) LSV of MoS_2 electrocatalytic assemblies (scan rate of 50 mV/s) performed in the operando cell for the electrochemical reduction of CO_2 in 1 M choline chloride and 1 M KOH (b) DEMS measurements of CO and H_2 production during LSV of MoS_2 electrocatalytic assemblies (scan rate of 1 mV/s), performed in a two-compartment cell.

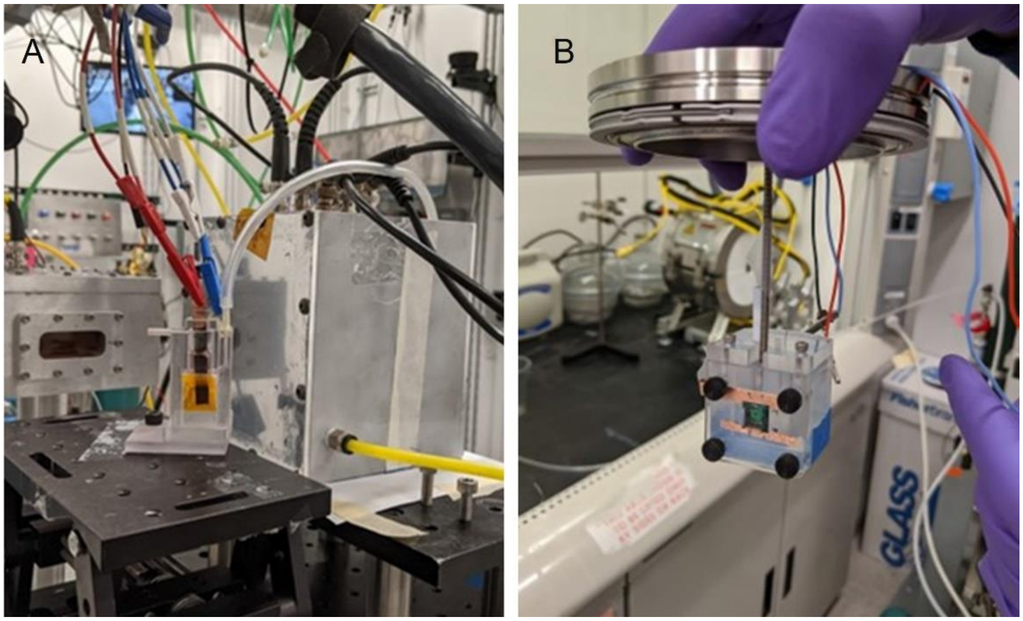

Figure S3: *Operando* cell used for XAS at the (a) Mo K- and (b) S K-edge.

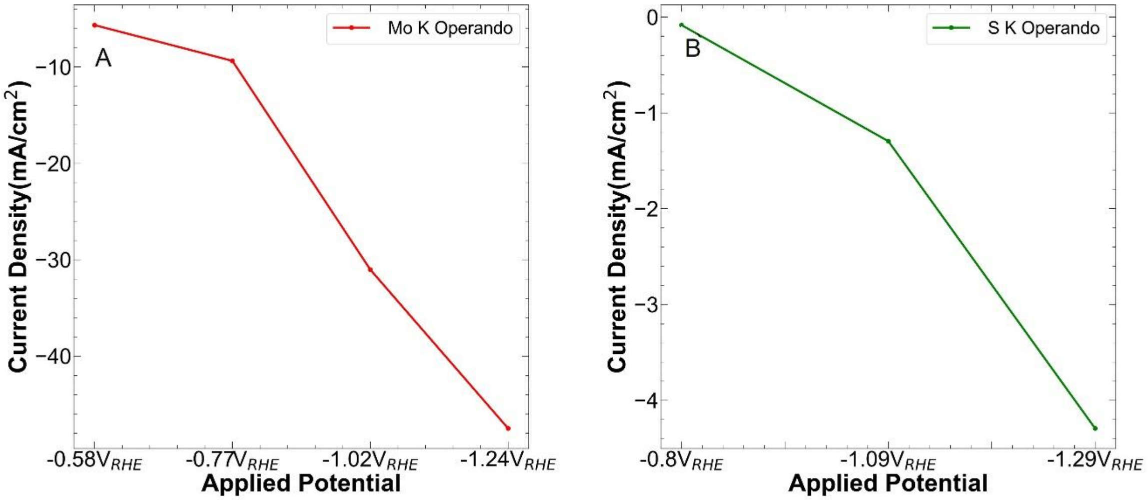

Figure S4: Current density collected under chronoamperometric conditions during *operando* XAS experiments at the (a) Mo K- and (b) S K-edge in their respective cells in **Figure S3**.

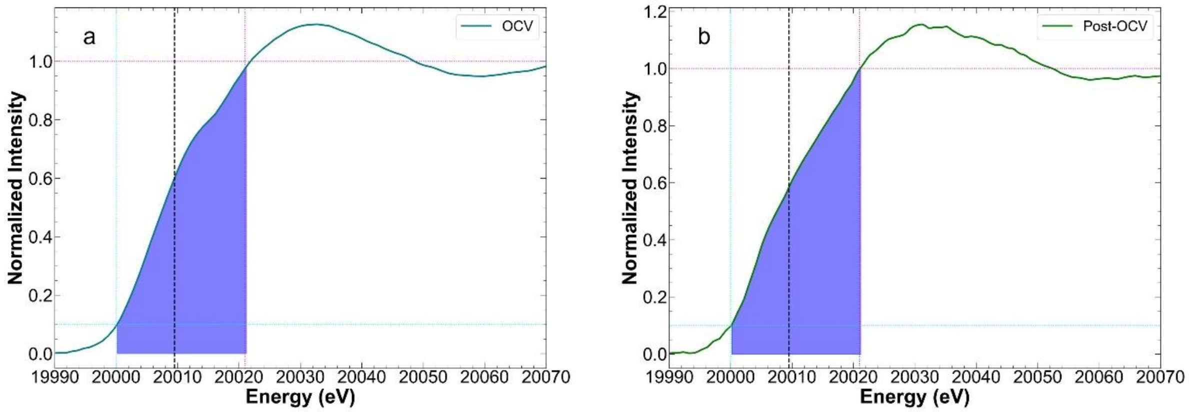

Figure S5: Example of the calculation of the position of the Mo K-edge, shown for OCV and post-OCV spectra. The calculated weighted average edge position is shown with vertical black line, whereas the shaded blue area denotes the data used in the calculation, bounded by μ_1= 0.1 and μ_2= 1.0.

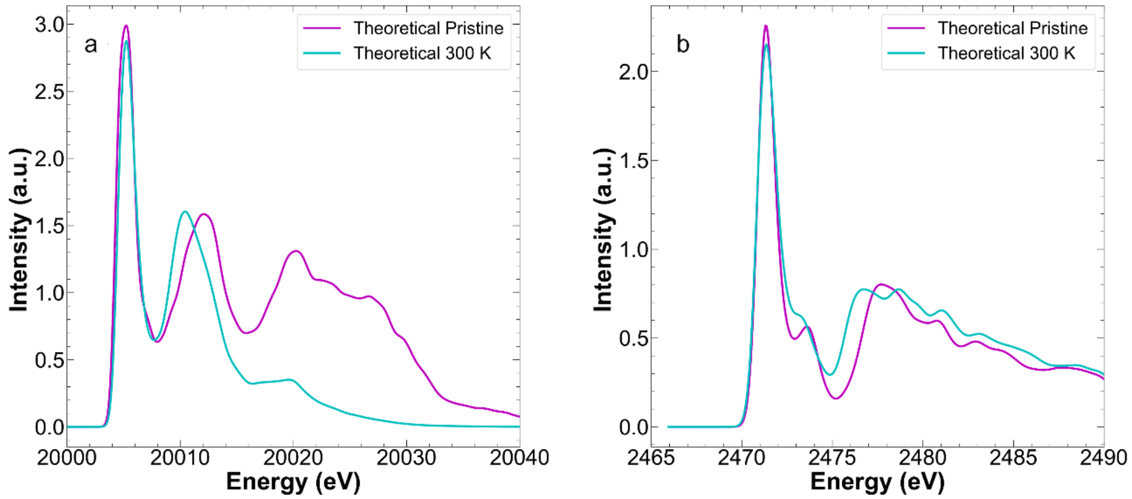

Figure S6: Simulated XAS of MoS_2 at the (a) Mo K-edge. We simulate the spectra by sampling an AIMD trajectory 300 K (green line) and compare to the XAS using the static crystal structure (purple line). We find large variability in the edge positions, indicating that the XAS is sensitive to the local environment. (b) Simulated S K-edge XAS obtained in the same conditions.

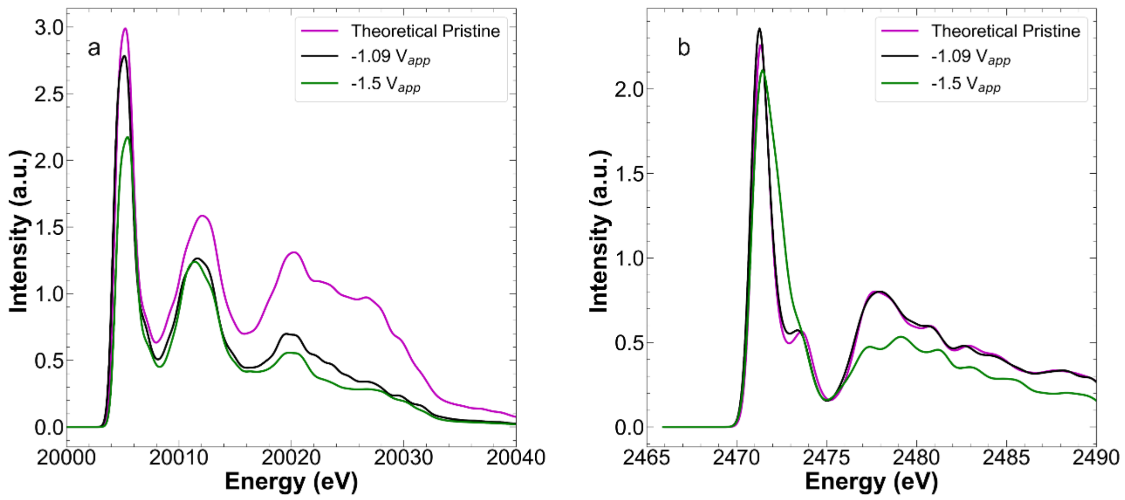

Figure S7: Changes to calculated (a) Mo K-edge and (b) S K-edge XAS with applied biased potential. The applied potential is referred versus a vacuum state.

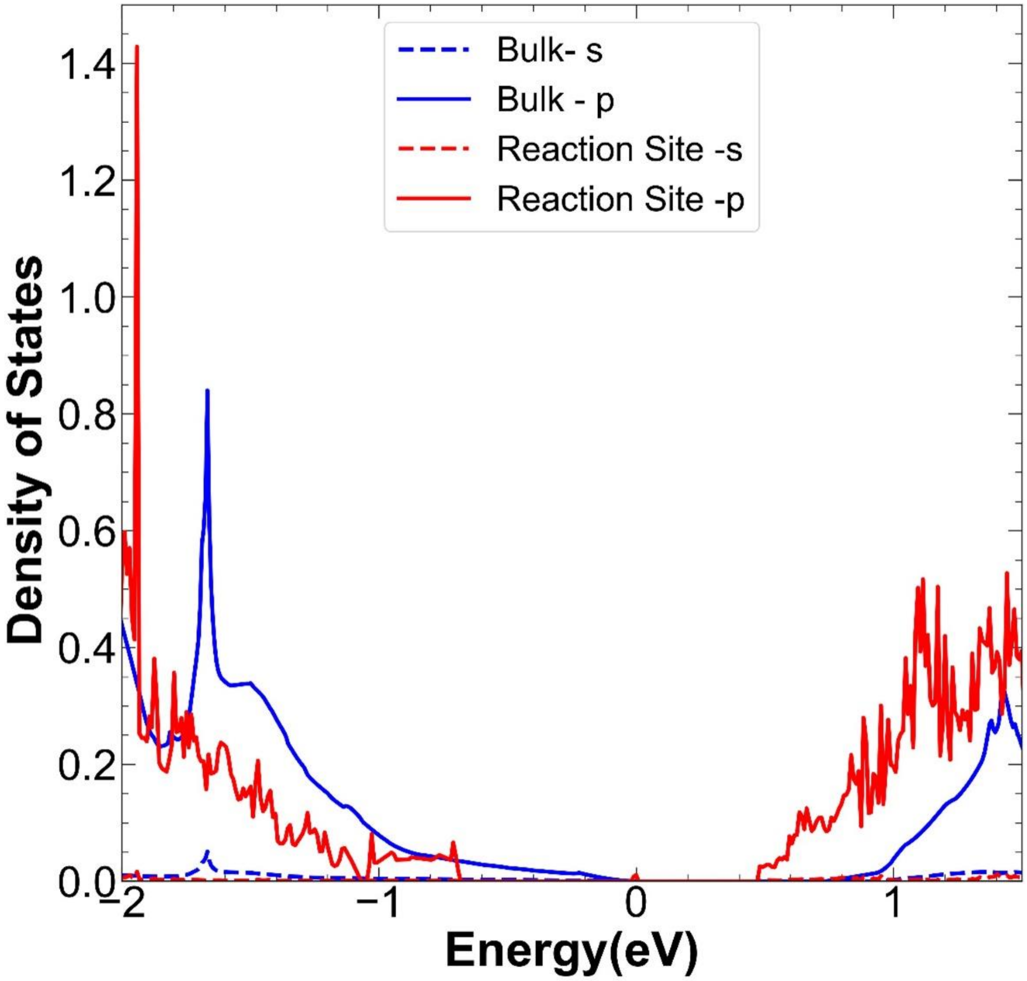

Figure S8: Comparison of the density of states of S atoms in the bulk of MoS_2 and the nearest neighbor to the reaction site where CO_2 binds to MoS_2.

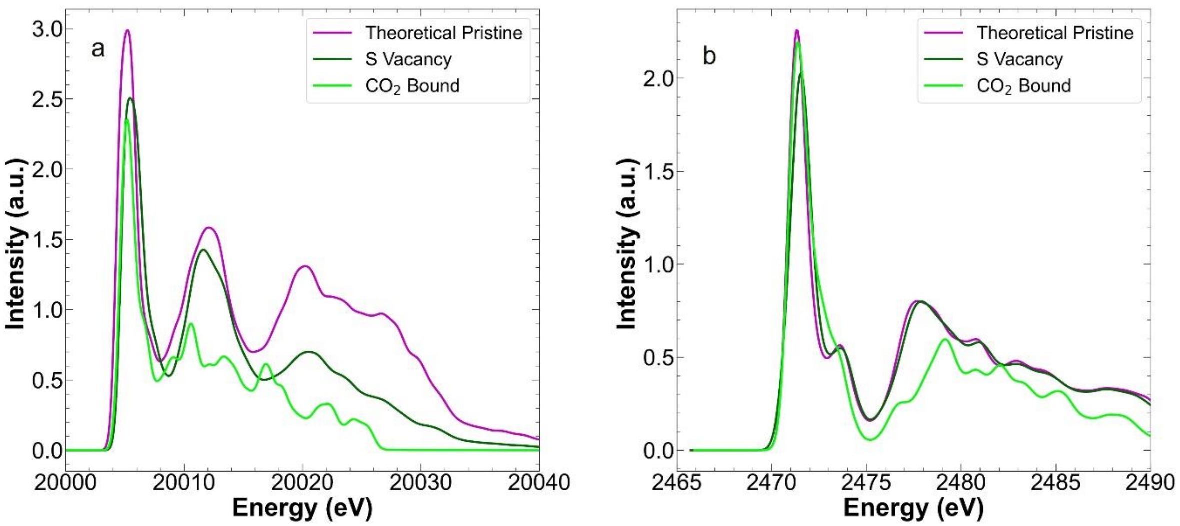

Figure S9: (a) Mo K- and (b) S K-edge XAS in the presence of single S vacancy and with bound CO_2.

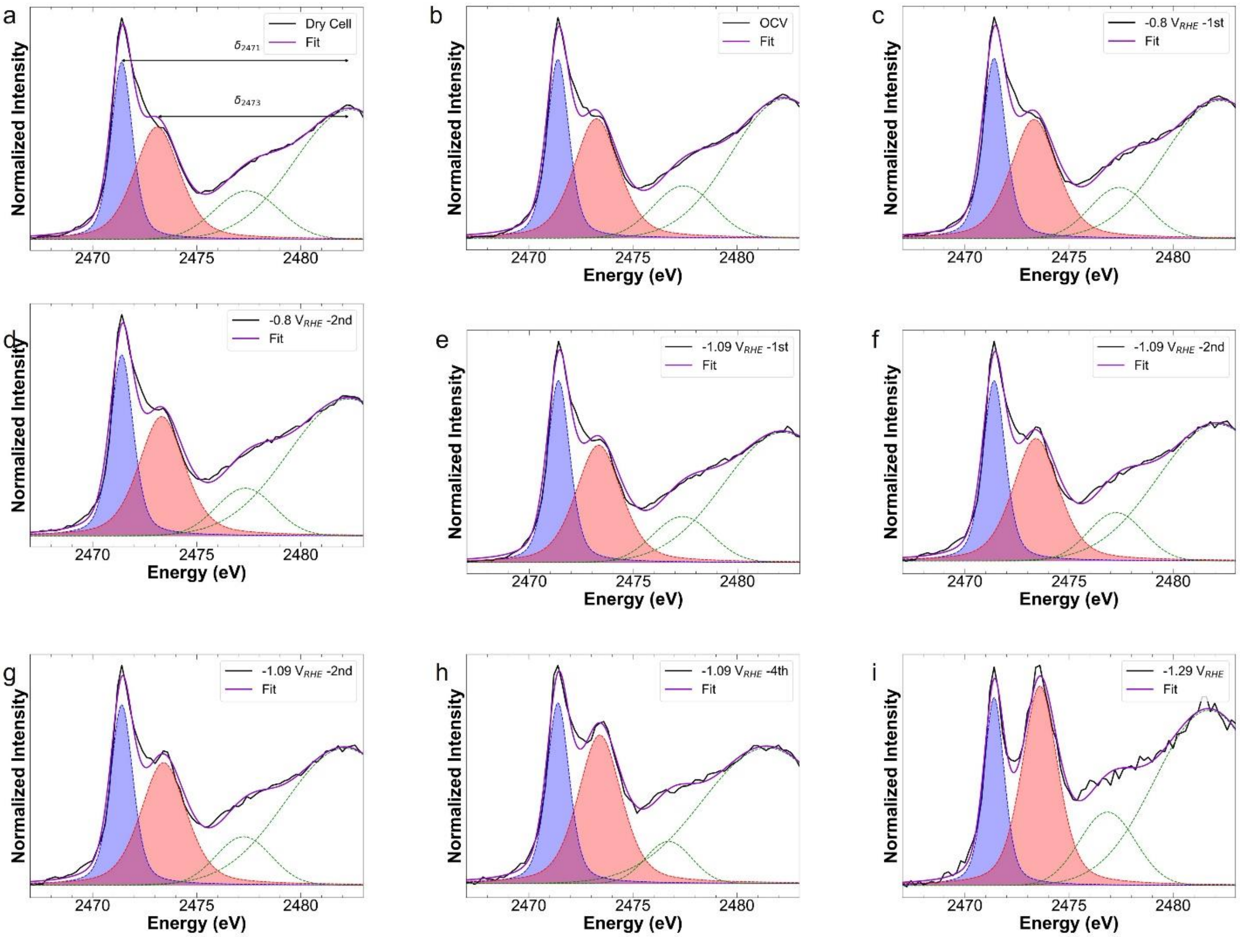

Figure S10: Results of fits of the S K-edge XAS.

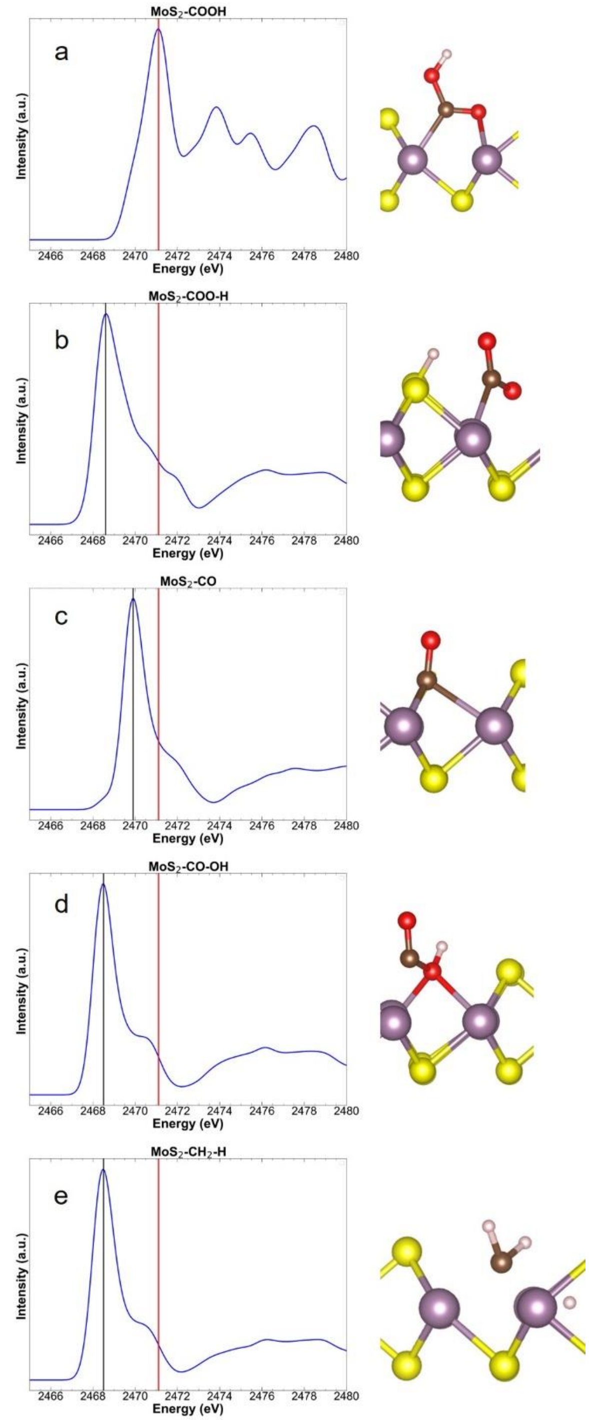

Figure S11: S K-edge of different MoS_2 reaction intermediates during CO_2 reduction a) -COOH, b) -COO-H, c) -CO, d) -CO-OH and e) $-CH_2$-H. The positions of the peak of other intermediates do not align with the experiment ruling them out from being observed *in-operando*

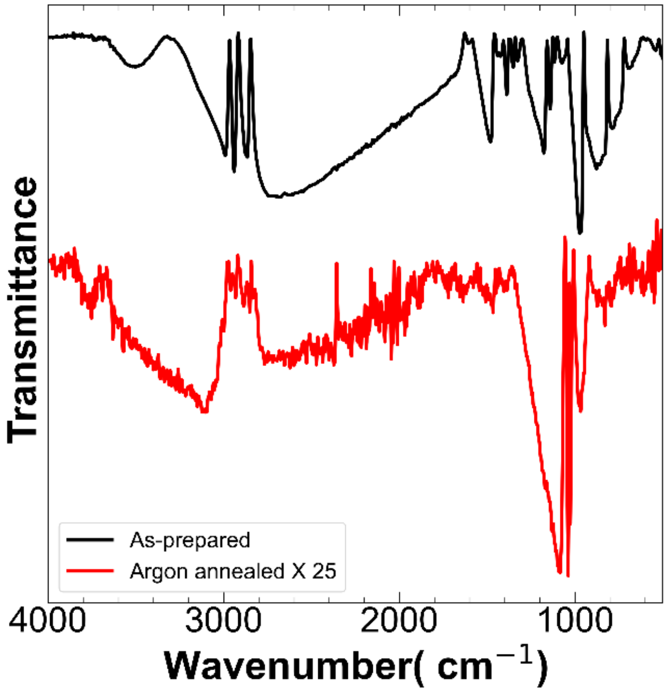

Figure S12: Infra-red spectrum of as-prepared MoS_2 compared to the product after annealing in Ar (magnified 25 times).

Supplementary References

(1) Altavilla, C.; Sarno, M.; Ciambelli, P. A novel wet chemistry approach for the synthesis of hybrid 2D free-floating single or multilayer nanosheets of MS_2@oleylamine (M═Mo, W). *Chem. Mater.* **2011**, *23*, 3879–3885.

(2) Ravel, B.; Newville, M. ATHENA, ARTEMIS, HEPHAESTUS: data analysis for X-ray absorption spectroscopy using IFEFFIT. *J. Synchrotron Radiat.* **2005**, *12*, 537–541.

(3) Newville, M.; Stensitzki, T.; Allen, D. B.; Ingargiola, A. LMFIT: Non-Linear Least-Square Minimization and Curve-Fitting for Python. Zenodo **2014**. https://doi.org/10.5281/ZENODO.11813.

(4) Yang, D.; Sandoval, S. J.; Divigalpitiya, W. M. R.; Irwin, J. C.; Frindt, R. F. Structure of single-molecular-layer MoS_2. *Phy. Rev. B* **1991**, *43*, 53–56.

(5) Du, R.; Shi, Z.; Yu, C.; Rao, W.; Xu, C.; Wang, J. Synthesis of graphene oxide/sulfur composites for advanced lithium-sulfur batteries. *Ionics* **2021**, *27*, 4269-4279.

(6) Xiang, T.; Fang, Q.; Xie, H.; Wu, C.; Wang, C.; Zhou, Y.; Liu, D.; Chen, S.; Khalil, A.; Tao, S.; Liu, Q.; Song, L. Vertical 1T-MoS_2 nanosheets with expanded interlayer spacing edged on a graphene frame for high rate lithium-ion batteries. *Nanoscale* **2017**, *9*, 6975–6983.

(7) Mourdikoudis, S.; Liz-Marzán, L. M. Oleylamine in nanoparticle synthesis *Chem. Mater.* **2013**, *25*, 1465–1476.

(8) Dau, H.; Liebisch, P.; Haumann, M. X-ray absorption spectroscopy to analyze nuclear geometry and electronic structure of biological metal centers-potential and questions examined with special focus on the tetra-nuclear manganese complex of oxygenic photosynthesis. *Anal. Bioanal. Chem.* **2003**, *376*, 562–583.

(9) Giannozzi, P.; Baroni, S.; Bonini, N.; Calandra, M.; Car, R.; Cavazzoni, C.; Ceresoli, D.; Chiarotti, G. L.; Cococcioni, M.; Dabo, I.; Dal Corso, A.; de Gironcoli, S.; Fabris, S.; Fratesi, G.; Gebauer, R.; Gerstmann, U.; Gougoussis, C.; Kokalj, A.; Lazzeri, M.; Martin-Samos, L.; Marzari, N.; Mauri, F.; Mazzarello, R.; Paolini, S.; Pasquarello, A.; Paulatto, L.; Sbraccia, C.; Scandolo, S.; Sclauzero, G.; Seitsonen, A. P.; Smogunov, A.; Umari, P.; Wentzcovitch, R. M. QUANTUM ESPRESSO: a modular and open-source software project for quantum simulations of materials. J. *Phys.: Condens. Matter* **2009**, *21*, 395502.

(10) Kohn, W.; Sham, L. J. Self-consistent equations including exchange and correlation effects. *Phys. Rev.* **1965**, *140*, A1133-A1138.

(11) Vanderbilt, D. Soft self-consistent pseudopotentials in a generalized eigenvalue formalism. *Phys. Rev. B* **1990**, *41*, 7892-7895.

(12) Perdew, J. P.; Burke, K.; Ernzerhof, M. Generalized gradient approximation made simple. *Phys. Rev. Lett.* **1996**, *77*, 3865-3868.

(13) Otani, M.; Sugino, O. First-principles calculations of charged surfaces and interfaces: A plane-wave nonrepeated slab approach. *Phys. Rev. B* **2006**, *73*, 115407.

(14) Kresse, G.; Hafner, J. Norm-conserving and ultrasoft pseudopotentials for first-row and transition elements. *J. Phys.: Condens. Matter,* **1994** *6*, 8245–8257

(15) Nosé, S. A unified formulation of the constant temperature molecular dynamics methods. *J.Chem. Phys* **1984**, *81*, 511.

(16) Monkhorst, H. J.; Pack, J. D. Special points for Brillouin-zone integrations. *Phys. Rev B* **1976**, *13*, 5188.

(17) Gunnarsson, O. and Lundqvist, B. I. Exchange and correlation in atoms, molecules, and solids by the spin-density-functional formalism*. *Phys. Rev. B* **1976**, *13*, 4274–4298.

(18) Prendergast, D.; Galli, G. X-ray absorption spectra of water from first principles calculations. *Phys. Rev. Lett.* **2006**, *96*, 215502.

(19) Liang, Y.; Vinson, J.; Pemmaraju, S.; Drisdell, W. S.; Shirley, E. L.; Prendergast, D. Accurate X-Ray spectral predictions: An advanced self-consistent-field approach inspired by many-body perturbation theory. *Phys. Rev. Lett.* **2017**, *118*, 096402.

(20) Kang, S.; Han, S.; Kang, Y. Unveiling electrochemical reaction pathways of CO_2 reduction to C_N species at S-vacancies of MoS_2. *ChemSusChem* **2019**, *12*, 2671–2678.

(21) Jiang, P.; Prendergast, D.; Borondics, F.; Porsgaard, S.; Giovanetti, L.; Pach, E.; Newberg, J.; Bluhm, H.; Besenbacher, F.; Salmeron, M. Experimental and theoretical

investigation of the electronic structure of Cu_2O and CuO thin films on Cu(110) using x-ray photoelectron and absorption spectroscopy. *J. Chem. Phys.* **2013**, *138,* 024704.

(22) Krause, P.; Sonk, J. A.; Schlegel, H. B. Strong field ionization rates simulated with time-dependent configuration interaction and an absorbing potential. *J. Chem. Phys.* **2014**, *140*, 174113.

(23) Grimme, S.; Antony, J.; Ehrlich, S.; Krieg, H. A A consistent and accurate *ab initio* parametrization of density functional dispersion correction (DFT-D) for the 94 elements H-Pu. *J. Chem. Phys.* **2010**, *132*, 154104.

(24) Huber, S. P.; Gullikson, E.; van de Kruijs, R. W. E.; Bijkerk, F.; Prendergast, D. Oxygen-stabilized triangular defects in hexagonal boron nitride. *Phys. Rev. B* **2015**, *92*, 245310.

(25) Komsa, H.-P.; Rantala, T. T.; Pasquarello, A. Finite-size supercell correction schemes for charged defect calculations. *Phys. Rev. B* **2012**, *86*, 45112.

(26) Henkelman, G.; Uberuaga, B. P.; Jónsson, H. A climbing image nudged elastic band method for finding saddle points and minimum energy paths. *J. Chem. Phys.* **2000**, *113*, 9901-9904.

(27) Henkelman, G.; Jóhannesson, G.; Jónsson, H. Methods for finding saddle points and minimum energy paths. *Theoretical Methods in Condensed Phase Chemistry* Springer, Dordrecht, **2002**, pp 269–302.
