# Supplementary material for: Active States During the Reduction of CO2 by a MoS2 Electrocatalyst
Source: J Phys Chem Lett. 2023 Mar 27;14(13):3222–9. doi: 10.1021/acs.jpclett.2c03942 (PMC10084464; doi:10.1021/acs.jpclett.2c03942)
Supplement: Supplementary file 3 — jz2c03942_si_003.pdf [file jz2c03942_si_003.pdf]

jz-2022-03942n.R1

Name: Peer Review Information for "Active States During the Reduction of CO_2 by a MoS_2 Electrocatalyst"

First Round of Reviewer Comments

Reviewer: 1
Comments to the Author
This manuscript describes an experimental operando XANES study of CO2 reduction on MoS2 nanosheets. The XANES is used to study the atomic and electronic structure around both the Mo and S atoms. The results of the experiment and theory are used to propose a 2 step process of S vacancy formation, and subsequent binding of CO2 near the S vacancy. Theoretical results (AIMD, XANES, and defect formation energy) qualitatively explain some of the trends in the spectra in terms of defect percentage, with defect formation more likely at lower bias voltages. The physics and chemistry is certainly interesting to a wide audience, and I believe the manuscript should be published with some changes as listed below:

1) The theoretical approach is only listed in the supplementary material. Perhaps a single sentence describing it with the appropriate reference would be good to add.

2) While the theoretical results roughly describe some of the qualitative behavior seen, the comparison to experiment is not great. In particular, the large broad peak seen in all of the experimental results at ~2782 eV is not reproduced very well by any of the candidate structures. Similarly, the high bias (-1.29V) experimental spectrum with a large double peak structure is not well reproduced. Can the authors discuss what might be causing this discrepancy?

3) In the main text, MoS2|WE is used without definition.

4) There are quite a few typos. The manuscript should be read carefully before being sent for production.

Reviewer: 2
Comments to the Author
In this manuscript, Kumar and co-workers investigate MoS2 nanosheets for electrochemical CO2 reduction using XAS and first principles simulations. They find the formation of S vacancies at lower potentials followed by CO2 adsorption and further reaction at higher reducing potentials. This work is interesting, well supported by experiments and theory and provides crucial insights into the mechanism for CO2RR on earth abundant MoS2 catalysts. I have a few comments that the authors can consider:

1. The authors have to provide details of the ESM simulations that were used to calculate the formation energies under different applied potentials. How was the SHE reference chosen?

2. Can the authors provide further insights on the role of reducing potentials in lowering the formation energy of S vacancies?

3. While the authors state that the production of S vacancies during synthesis can help reduce overpotentials for CO2RR, such vacancies also lead to enhanced HER activity (ref 18). Therefore, the correlation between S vacancies and CO2RR activity is quite complicated. A comment in this regard would be appreciated.

4. The authors are requested to provide coordinates of the structures used for the XAS simulations (Figure 2) and in Figures 3 b,c to ensure reproducibility.

Author's Response to Peer Review Comments:

Reviewer(s)' Comments to Author:

Reviewer: 1

We thank the reviewer for your review of our work and for appreciating the significance. We address your concerns below. Your comments are reproduced verbatim in *italics* while our response follows in red. Where appropriate, we will indicate any major changes to the manuscript or SI by underline.

Recommendation: This paper is publishable subject to minor revisions noted. Further review is not needed.

Comments:
This manuscript describes an experimental operando XANES study of CO2 reduction on MoS2 nanosheets. The XANES is used to study the atomic and electronic structure around both the Mo and S atoms. The results of the experiment and theory are used to propose a 2 step process of S vacancy formation, and subsequent binding of CO2 near the S vacancy. Theoretical results (AIMD, XANES, and defect formation energy) qualitatively explain some of the trends in the spectra in terms of defect percentage, with defect formation more likely at lower bias voltages. The physics and chemistry is certainly interesting to a wide audience, and I believe the manuscript should be published with some changes as listed below:

1) The theoretical approach is only listed in the supplementary material. Perhaps a single sentence describing it with the appropriate reference would be good to add.

We have modified the text in the manuscript to now read:

"To further elucidate the physics behind the observed spectral changes, we simulated the operando XAS using snapshots obtained from AIMD simulations at 300K. Here, we employed density functional theory (DFT) to simulate the electronic structure of different MoS2 intermediates, coupled with the effective screening media method (ESM) to simulate the electrochemical effects. The XAS simulation were performed within a multi-determinant, delta self-consistent field approach (12-18), Further simulation details can be found in the supplementary material."

2) While the theoretical results roughly describe some of the qualitative behavior seen, the comparison to experiment is not great. In particular, the large broad peak seen in all of the experimental results at ~2782 eV is not reproduced very well by any of the candidate structures. Similarly, the high bias (-1.29V) experimental spectrum with a large double peak structure is not well reproduced. Can the authors discuss what might be causing this discrepancy?

We thank the reviewer for the question. The apparent disagreement between the simulations and experiments, especially in the line shape beyond the adsorption edge are due to difference between the methods used to procure the spectra, so that an expectation of a direct match in the intensities is not expected. We will detail these presently, however we note that any errors leading to such discrepancies are of the same magnitude. This allows us to quantitatively compare the spectral changes across different structures such as capturing the trend in Figure 3a.

In the experiments, the XAS spectra background subtraction is done from a line regressed from the before-edge region with a polynomial regression in the post-edge region. This approach, while standardly applied in the community, introduces some uncertainty, and sometimes flattening, in the relative intensity of the post-edge features. In the simulations, the higher energy features are generated non self-consistently, assuming the same potential as the 1st excited state. While this approximation makes our calculations tractable, it can lead higher energy peaks that are less intense than would be expected. We have included this as a discussion point in the manuscript, which reads:
"… suggesting that charging alone does not significantly alter the unoccupied states of MoS2. We note that any discrepancies between experimental and simulated spectra intensities can result from several factors, including the limited ability to normalize the experiments, due to the requirement for background subtraction, whereas the calculation does not have any background signal. Nevertheless, in our computational approach, these differences are self-contained, which allows us to quantitively compare spectra changes across different structures, producing relative intensities that compare well with experiments."

3) In the main text, MoS2|WE is used without definition.
We have updated the text in the manuscript to now read:
"…without electrolyte on the working electrode (referred as MoS2|WE)"

4) There are quite a few typos. The manuscript should be read carefully before being sent for production.
Thank you for the comment, we have updated the text and believe that all the typos are corrected.

Additional Questions:
Urgency: High

Significance: High

Novelty: High

Scholarly Presentation: High

Is the paper likely to interest a substantial number of physical chemists, not just specialists working in the authors' area of research?: Yes

Reviewer: 2

We thank the reviewer for your review of our work. Your comments were very helpful in improving the overall quality of the manuscript. We address your concerns below. Your comments are reproduced verbatim in *italics* while our response follows in red. Where appropriate, we will indicate any major changes to the manuscript or SI by underline.

Recommendation: This paper may be publishable, but major revision is needed; I would like to be invited to review any future revision.

Comments:
In this manuscript, Kumar and co-workers investigate MoS2 nanosheets for electrochemical CO2 reduction using XAS and first principles simulations. They find the formation of S vacancies at lower potentials followed by CO2 adsorption and further reaction at higher reducing potentials. This work is interesting, well supported by experiments and theory and provides crucial insights into the mechanism for CO2RR on earth abundant MoS2 catalysts. I have a few comments that the authors can consider:

1. The authors have to provide details of the ESM simulations that were used to calculate the formation energies under different applied potentials. How was the SHE reference chosen?

We thank the reviewer for the comment. In our computational approach, the applied potentials are defined in an absolute sense to the pristine system, without any need to refer to an reference electrode (such as the SHE). We have expanded section 18 in the SI to include detail of ESM methods. The section now reads:

"We employed the effective screening media method (ESM) to simulate an MoS2 electrode under applied bias. The effective screening media method is a framework developed for first-principles simulating of electrified interfaces, where the system Hamiltonian includes an additional term representing the interaction between electronic charge and an imaginary dielectric screening medium. Thus, a specific bias potential can be simulated by a Green's function of the charge (the charge – number of electrons – and the potential are conjugate pairs). The applied voltage employed in the simulation is the result of electrostatic potential difference between MoS2 and the ESM."

2. Can the authors provide further insights on the role of reducing potentials in lowering the formation energy of S vacancies?

We thank the reviewer for the question. In general, the presence of vacancies leads to the formation of midgap states, and the application of a negative bias (i.e., excess electrons) could potentially fill up these states. Another effect is that the additional electron density near the fermi

level at negative bias reduces the (original pristine) MoS2 LUMO. The net result is that energy of forming the vacancy sites is stabilized at lower potentials. Mathematically, this process can be understood from the equation in S20, where since q is negative and increasing fermi energy (E_f) leads to an overall decrease in the formation energy.

We elaborate this point in the manuscript:

"For instance, the formation energy for the structure with 4% defects lowered from 2eV at OCV to ~ 1 eV at -1.09 eV, to ~ 0.5 eV at -1.29 eV (**Fig. 3b**). The stabilization of the defects at increasingly negative bias results from the eventual filling of any new mid-gap states and extra electrostatic screening due to the injection of excess electrons, which overall lowers the cost of vacancy formation."

3. While the authors state that the production of S vacancies during synthesis can help reduce overpotentials for CO2RR, such vacancies also lead to enhanced HER activity (ref 18). Therefore, the correlation between S vacancies and CO2RR activity is quite complicated. A comment in this regard would be appreciated.

We thank the reviewer for this comment. We note that our work is in agreement with recent literature, including by some of us, where CO_2RR activity in MoS_2 is promoted by the presence of S vacancies. Additionally, we note that CO was the major product in the applied potential range used in the *operando* experiments (Fig S2 b).
We have modified the manuscript to further elaborate the point:
"with increased bias voltage, from > 6 eV at OCV to < 2 eV at -1.29 eV (Fig. 3c). Increase in stability of these vacancy sites as the active site for CO_2 binding and favorable pathway due to negative potential together promote greater CO_2RR. Our calculation shows good agreement with recent work where basal plane MoS_2 S vacancies are favorable to CO_2RR.[ref 41] ".

4. The authors are requested to provide coordinates of the structures used for the XAS simulations (Figure 2) and in Figures 3 b,c to ensure reproducibility.

We have included the structures used for theoretical calculations from Fig. 3b,3c. For convenience, the files are in POSCAR format as it contains both coordinates and lattice parameters.

Additional Questions:
Urgency: High

Significance: High

Novelty: High

Scholarly Presentation: High

Is the paper likely to interest a substantial number of physical chemists, not just specialists working in the authors' area of research?: Yes

jz-2022-03942n.R2

Name: Peer Review Information for "Active States During the Reduction of CO_2 by a MoS_2 Electrocatalyst"

Second Round of Reviewer Comments

Reviewer: 2

Comments to the Author
The authors have provided satisfactory responses to my comments and I recommend publication.

Reviewer: 1

Comments to the Author
After reviewing the resubmitted manuscript, all of my comments have been addressed. At this point, the manuscript is suitable for publication.

Author's Response to Peer Review Comments:

February 22, 2023

Prof. Editor
The Journal of Physical Chemistry Letters
Re: Revisions for Manuscript ID jz-2022-03942n.R1
Title: Active states during the reduction of CO2 by a MoS2 electrocatalyst
Authors: Khagesh Kumar, Sasawat Jamnuch, Leily Majidi, Saurabh Misal, Alireza Ahmadiparidari, Michael A. Dato, George E. Sterbinsky, Tianpin Wu, Amin Salehi-Khojin, Tod A. Pascal and Jordi Cabana

Dear Prof. Editor,
On Feb 21st you wrote:
"*Dear Dr. Pascal:*

Thank you for submitting your revised manuscript. We are ready to accept your submission after the following non-scientific changes:"

We are delighted that our work has been accepted and would again like to express our appreciation to you and the reviewers for strengthening our manuscript. We have made the following editorial changes, as recommended in your decision letter:

1. We have uploaded "clean" versions of the manuscript and supplementary materials, without any markups
2. We have resized the TOC graphic and included it after the abstract in the main text
3. We have removed "S" from the page numbers in the main text
4. We have decided to not try for the cover art

Thank you again for considering and accepting our work

Sincerely,

Tod A Pascal, corresponding author, on behalf of all the authors
